# 1123. Case Study. Adhesive Capsulitis as a Rare Complication of Large Upper Extremity Burn: A Case Report

**DOI:** 10.1093/jbcr/irag033.526

**Published:** 2026-04-06

**Authors:** Mare G Kaulakis, Sarah M Tepe, Christopher J Fedor, Francesco M Egro

**Affiliations:** University of Pittsburgh Medical Center, Mercy Burn Center, Pittsburgh, PA; University of Pittsburgh Medical Center, Pittsburgh, PA; University of Pittsburgh Medical Center, Department of Plastic Surgery, Pittsburgh, PA; University of Pittsburgh Medical Center, Pittsburgh, PA

## Abstract

**Patient Presentation (age range, injury details, relevant history):**

We present a case of a patient with extensive left upper extremity, trunk and back burns who developed contracture along with adhesive capsulitis requiring surgical intervention by multiple specialties. The patient is a 76-year-old male who sustained 17% TBSA mixed partial and full thickness flame burns to the left upper extremity, trunk and back that required multiple excisions and skin grafting procedures during the initial hospitalization.

**Clinical Challenges:**

The patient developed severe banding of the left anterior and posterior axillary lines which limited his range of motion significantly. This deficit greatly impacted his quality of life and required multiple months of physical and occupational therapy in attempt to regain some functionality of his left upper extremity.

**Management Approach:**

He underwent a staged contracture release consisting of Z-plasty adjacent tissue transfer to left axilla and back, multiple CO2 laser treatments and a pedicled left latissimus dorsi muscle flap with autograft. He was referred to orthopedic surgery after an x-ray noted severe osteoarthritis of his left shoulder and was ultimately diagnosed with adhesive capsulitis. The patient was taken to the operating room with Orthopedic Surgery for capsule release which yielded improved range of motion. Due to persistent discomfort and weakness of the left upper extremity, the patient recently underwent steroid injections in clinic. He was seen by Plastic Surgery, now 1 month after capsule release, and has an excellent surgical result.

**Outcomes:**

The patient has continued to exhibit excellent surgical result with increased range of motion in the left upper extremity after contracture release and capsule release performed by Plastic and Orthopedic surgery, respectively.

**Lessons Learned:**

Adhesive capsulitis is a rare but clinically significant complication of major burn injury. Distinguishing this entity from burn scar contracture is essential, as management strategies differ and may include intra-articular therapies rather than solely cutaneous release. Early recognition can guide appropriate treatment and optimize functional recovery in burn survivors.

**Applicability to Practice:**

This case highlights the collaborative effort between Burn, Plastic and Orthopedic surgery in order to treat a patient with severe burns.

